# Oligodendroglial primary cilium heterogeneity during development and demyelination/remyelination

**DOI:** 10.3389/fncel.2022.1049468

**Published:** 2022-11-24

**Authors:** Giada Delfino, Karelle Bénardais, Julien Graff, Brigitte Samama, Maria Cristina Antal, M. Said Ghandour, Nelly Boehm

**Affiliations:** ^1^ICube Laboratory UMR 7357, Team IMIS, Strasbourg, France; ^2^Institut d’Histologie, Service Central de Microscopie Electronique, Faculté de Médecine, Université de Strasbourg, Strasbourg, France; ^3^Fédération de Médecine Translationnelle de Strasbourg (FMTS), Strasbourg, France; ^4^Hôpitaux Universitaires de Strasbourg, Strasbourg, France

**Keywords:** OPCs, primary cilium, development, myelination, demyelinating diseases

## Abstract

The primary cilium (PC) has emerged as an indispensable cellular antenna essential for signal transduction of important cell signaling pathways. The rapid acquisition of knowledge about PC biology has raised attention to PC as a therapeutic target in some neurological and psychiatric diseases. However, the role of PC in oligodendrocytes and its participation in myelination/remyelination remain poorly understood. Oligodendrocyte precursor cells (OPCs) give rise to oligodendrocytes during central nervous system (CNS) development. In adult, a small percentage of OPCs remains as undifferentiated cells located sparsely in the different regions of the CNS. These cells can regenerate oligodendrocytes and participate to certain extent in remyelination. This study aims characterize PC in oligodendrocyte lineage cells during post-natal development and in a mouse model of demyelination/remyelination. We show heterogeneity in the frequency of cilium presence on OPCs, depending on culture conditions *in vitro* and cerebral regions *in vivo* during development and demyelination/remyelination. *In vitro*, Lithium chloride (LiCl), Forskolin and Chloral Hydrate differentially affect cilium, depending on culture environment and PC length correlates with the cell differentiation state. Beside the role of PC as a keeper of cell proliferation, our results suggest its involvement in myelination/remyelination.

## Introduction

In the last two decades, a century-forgotten organelle, the primary cilium (PC), has emerged as an indispensable cellular antenna, involved in several cell functions. PC forms when a cell has completed mitosis and enters G0/G1 phase (Hsu et al., 2017; Wang and Dynlacht, 2018). PC is essential for transduction of numerous important cells signaling pathways, particularly Sonic hedgehog (Shh) (Corbit et al., 2005). In the central nervous system (CNS), PC has been largely studied in neurons (Baudoin et al., 2012; Bae et al., 2019; Toro-Tapia and Das, 2020), which possess a PC from neuroepithelial to post-mitotic stage. During brain development, PC affects neuronal precursors migration and differentiation, and the ablation of PC impairs interneurons migration from ganglionic eminence to the dorsal cortex (Higginbotham et al., 2012). In adult, PC seems to play a role in neuronal energy metabolism (Han et al., 2014; Song et al., 2018) and learning and memory cognitive functions (Berbari et al., 2014).

For long time, ciliary disfunctions have been correlated exclusively with the pathogenesis of ciliopathies. In a previous study, we showed the presence of one of the Bardet-Biedl syndrome proteins, in oligodendrocytes lineage in human and mouse CNS (Bénardais et al., 2021). Recent studies now point at PC implication in other pathologies such as neurological and psychiatric diseases (Hu et al., 2017; Muñoz-Estrada et al., 2018; Bae et al., 2019; Mustafa et al., 2019). A correlation between cilium length and cellular functions has been observed in some cell types. For examples, elongated PC in hippocampal neurons, through overexpression of serotonin 5-HT6 receptor at PC membrane, has been correlated with cognition impairment in APP/PS1 mouse (Hu et al., 2017). Also, in striatal neurons of a mouse model of Huntington’s disease, shorter PC were correlated with age progression and mHTT (mutant Huntingtin) accumulation (Mustafa et al., 2019).

PC length adapts to environmental cues or drug treatments. Lithium Chloride (LiCl), a well-known treatment for bipolar disease is a potent PC elongation drug. LiCl elongates PC in different cell types, such as neurons (Miyoshi et al., 2009), synoviocytes, astrocytes (Ou et al., 2009), fibroblasts (Nakakura et al., 2015), chondrocytes (Thompson et al., 2016), or osteoblasts (Oliazadeh et al., 2017). In human, PC in olfactory neurons precursors cells obtained from schizophrenia and bipolar disorders patients were elongated in Lithium treated patients (Muñoz-Estrada et al., 2018). Several intracellular pathways have been implicated in cilium length control, among them cAMP pathway; for example, cAMP and forskolin elongate PC in renal (Sherpa et al., 2019) or endothelial cells (Besschetnova et al., 2010; Abdul-Majeed et al., 2012), although cAMP elevation induced cilia resorption in an embryonic renal cell line (Porpora et al., 2018). Ablation of PC by Chloral Hydrate has also been used to study correlation between PC and cell function (Deren et al., 2016; Martín-Guerrero et al., 2020; Shi et al., 2020; Wei et al., 2022).

The acquisition of knowledge about PC biology in some cell types during the past years has raised the possibility that PC might be a therapeutic target in some diseases (Arrighi et al., 2017; Spasic and Jacobs, 2017; Wang et al., 2021). However, the role of PC in oligodendroglial cells remains poorly understood and particularly in relation to myelination/remyelination. In myelin degenerating diseases, such as multiple sclerosis, enhancing regeneration of CNS myelin is an important therapeutic goal (Franklin and Ffrench-Constant, 2017). Indeed, chronic loss of myelin will lead to axonal and neuronal degeneration. Preventing neurodegeneration may be achieved by directly targeting the neurons or by accelerating remyelination. Remyelination following oligodendrocytes loss relies on oligodendrocyte precursor cells (OPCs) either deriving from quiescent resident OPCs (Franklin and Ffrench-Constant, 2008; Zawadzka et al., 2010) or from subventricular neural progenitor cells (Nait-Oumesmar et al., 2007; Xing et al., 2014; Kang et al., 2019) which migrate and differentiate in the damaged area. Heterogeneity in OPCs cells has appeared in lasts years (Bribián et al., 2020; Beiter et al., 2022; Hilscher et al., 2022); a spectrum of OPCs phenotypes depending on age (Spitzer et al., 2019), transcriptional factors (Beiter et al., 2022), environmental cues (Bribián et al., 2020), and regional localization (Hilscher et al., 2022) has been described. Recent studies report the presence of a PC in OPCs that disappears concomitantly with cells differentiation (Falcon-Urrutia et al., 2015; Cullen et al., 2020). The present work aims at studying the status of oligodendrocyte PC during oligodendrocytes development and in a mouse model of demyelination/remyelination in relation with their microenvironment and anatomical localization.

## Materials and methods

### Animals

The *plp*-eGFP transgenic mice expressing the enhanced green fluorescent protein (eGFP) driven by the myelin proteolipid protein gene (*plp*) promoter in C57BL/6 genetic background were generously provided by Dr. W. Macklin (Cleveland Clinic Foundation, Ohio, USA) and housed in the central animal facility of the Faculty of Medicine in Strasbourg. Animals were maintained under fixed 12 h light/dark cycle with free access to water and food. All procedures were conducted in accord with the guidelines for animal care and the experimental protocol was approved by the local ethics committee (CREMEAS, reference n° #17089-2018101116367904 v2).

### Mouse purified oligodendrocyte precursor cells cultures

Mixed primary glial cell cultures were prepared according to O’Meara et al. (2011) with slight modifications. Briefly, post-natal day 0 (P0) *plp*-eGFP pups were killed by decapitation and cerebral hemispheres were isolated, diced and digested in 0.3% Papain, 0.3% L-cysteine and 0.06% DNase I at 37°C for 20 min. Enzymatic reaction was stopped by DMEM (1X) Glutamax medium (Gibco, France) supplemented with 10% fetal bovine serum (FBS) (Gibco, France) and 0.5% penicillin/streptomycin. Cells were dissociated, filtered through a 40μm nylon cell strainer, and centrifuged at 1,200 rpm. The pellet was resuspended in supplemented DMEM (1X) Glutamax and cells were plated into 100 mm Petri dishes previously coated with poly-L-lysine and placed at 37°C in humified 5% CO_2_ incubator for 12 days. OPCs grown on the bed layer of astrocytes were mechanically dissociated, the medium was collected, replated in uncoated Petri dish, and incubated at 37°C in humified 5% CO_2_ for 30 min. After incubation, the medium was collected, centrifuged at 1,200 rpm and the pellet was resuspended in proliferation or differentiation medium. Proliferation and differentiation media components are listed in Supplementary Table 1. Cells were plated on poly-L-lysine coated coverslips at 5 × 10^4^ per well into 24-well plates and grown in the same incubation conditions as above during 24, 48 and 72 h (1, 2, 3 DIV).

### Oligodendroglial cell line 158N culture

The immortalized murine oligodendroglia cell line 158N (Feutz et al., 1995) was cultured on poly-L-lysine coated coverslips in DMEM (1X) Glutamax medium supplemented with 10% FBS and 0.5% penicillin/streptomycin in an incubator at 37°C in humified 5% CO_2_ until 70% confluence was reached.

### Cell culture treatments

Purified OPCs cultures and cell line 158N were treated for 20 h with 10 mM of LiCl (Sigma, France) or 50 μM forskolin (Sigma, France) dissolved, respectively, in proliferation or differentiation medium or in serum free DMEM (1X) Glutamax. To study primary cilium ablation, purified OPCs were treated for 20 h with or without 2 mM Chloral Hydrate dissolved in proliferation or differentiation medium and fixed at 2 DIV.

### Cuprizone induced demyelination mouse model

*Plp*-eGFP mice, 8 weeks old, were used to study oligodendrocytes PC during demyelination.

At the time of weaning, experimental mice were placed randomly four per cage and fed with a normal chow. At 8 weeks old, control and treated mice were assigned randomly to their experimental group and weighed. The mean weight of mice was 20 g.

Cuprizone 0.2% (bis-cyclohexanone-oxaldihydrazone; Sigma, France) was mixed with milled chows and added to feeders each day (5–8 g per mouse). Animals were treated with or without 0.2% of cuprizone for 3 or 6 weeks; a last group of animals was treated or not for 6 weeks with cuprizone and euthanized 6 weeks after treatment withdrawal.

### Immunocytochemistry

Freshly prepared 4% formaldehyde in 0.1 M phosphate buffer pH 7.4 was used as the fixative. Cells were fixed for 24 h. P2 mice were anesthetized by ice, all the others were anaesthetized by ketamine (8 mg/kg)/Xylazine (5–16 mg/kg) and fixed by transcardial perfusion. Brains were post-fixed for three days in the same fixative and then coronal 50 or 20 μm sections were prepared on a vibratome and processed as free-floating sections.

Antibodies were diluted in blocking solution (2% normal horse serum in PBS containing 0.2% of Triton X-100). For single staining, cells or sections were exposed to primary antibodies diluted in blocking solution overnight at room temperature, then washed in PBS/0.2% of Triton X-100 and incubated for 2 h in appropriate secondary antibodies. Biotinylated antibody reaction was revealed using peroxidase-labeled streptavidin complex (Vectastain Elite kit, Vector Laboratories, Abcys, France) followed by VIP, SG (Vector Laboratories, Abcys, France) or HistoGreen (Novus Biologicals, France) as peroxidase substrates.

When double immunofluorescence was performed using antibodies from two different species (mouse/rabbit), cells were exposed to the mix of primary antibodies overnight and then incubated for 2 h in appropriate secondary antibodies.

For double staining using a fluorochrome and a chromogen or when the two primary antibodies derived from the same species, immunocytochemistry was completed first using a chromogen followed by a second immunostaining using a fluorochrome or a chromogen—tagged secondary antibody.

Mounting medium with Dapi (Vectashield, Vector Laboratories, Abcys, France) was used for fluorescence-labeled sections or cultures. Chromogen-revealed sections were mounted with Eukitt. The list of antibodies used in this study is accessible in Supplementary Tables 2, 3.

### Electron microscopy

158N cells were detached by a non-enzymatic cell dissociation solution (Ref. S-014-M, Sigma) and the pellet was fixed in 2.5% glutaraldehyde in cacodylate buffer, post-fixed in osmium tetroxyde and embedded in epon.

Ultrathin sections were stained with uranyl acetate and lead citrate and observed in a Phillips M208 electron microscope.

### Primary cilium length measurement

For the measurement of PC length, images were captured with a BX60 microscope equipped with DP70 digital camera (Olympus). For each cilium, three z-stacks were captured with 100X objective to analyze the full axoneme. Length was measured using ImageJ software (Image J, US National Institutes of Health, Bethesda, MD, USA).

### Study of oligodendrocyte precursor cells cilium during development and demyelination/remyelination

For developmental study, four groups of three male *plp*-eGFP mice were used at P2, P10, P15, P30. For demyelination experiments, six animals for each condition were analyzed.

All analyses were performed in three coronal sections located between Bregma 0.98 mm and Bregma −0.46 mm (Franklin and Paxinos atlas) from each animal. Cortex was segmented in two regions, a superficial region, corresponding to layers I, II and III, and a deep region, corresponding to layers IV, VI. Only the middle region of the corpus callosum was studied.

Images were captured with 20X objective on a light microscope (Coolpix 995, Nikon, France) for cells counting or with a 100X objective on fluorescent microscope (BX60, Olympus, France) to visualize PC.

OPCs, oligodendroglial cells and myelin were characterized respectively by PDGFR-a, OLIG2 and MBP immunostaining. Proliferating OPCs and ciliated OPCs were detected by double staining Ki67/PDGFR-a and ARL13b/PDGFR-a, respectively. Astrocytic and microglial reactions were determined, respectively, by GFAP^+^ and Iba1^+^ immunoreactivity. Bilateral areas for each section were photographed and analyzed using ImageJ software (Image J, US National Institutes of Health, Bethesda, MD, USA; see text footnote 1).

### Statistical analysis

**FIGURE 1:**
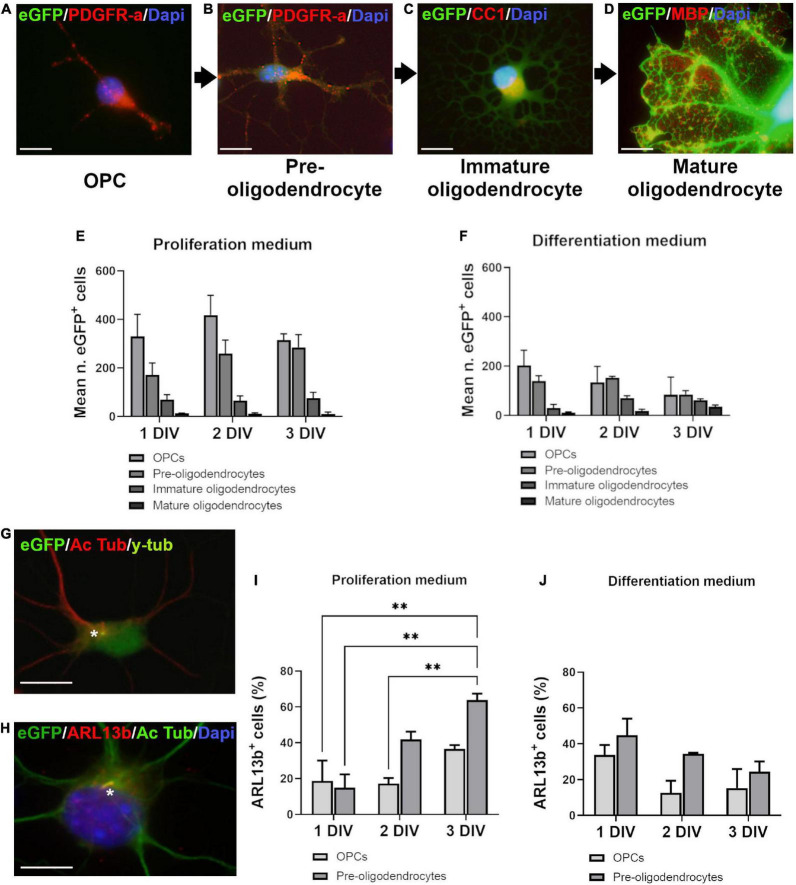
Ciliated cells in proliferation and differentiation media. **(A–D)** Oligodendroglial cells in culture were classified in four stages according to branching development, eGFP intensity and markers expression. PDGFR-a^+^ cells were divided in two stages: very weakly fluorescent bipolar cells (OPCs) **(A)** and more intensely fluorescent multipolar cells with short processes (pre-oligodendrocytes) **(B)**. Immature oligodendrocytes were CC1^+^/MBP^–^, intensely fluorescent highly branching cells **(C)**. The late stage cells were brightly fluorescent mature oligodendrocytes characterized by large flat membrane sheets, MBP^+^ **(D)**. **(E,F)** Distribution of eGFP^+^ cells in proliferation and differentiation media at three times of culture (days *in vitro*: DIV). Statistical significance was determined by Chi-Square Test (χ^2^). Proliferation medium: *df* 83.26, *p* < 0.001***; Differentiation medium: *df* 242.9, *p* < 0.001***. Graphs in **(E,F)** represent de mean number of eGFP^+^ cells analyzed in three coverslips from three independent experiences for each time. **(G,H)** Representative images of OPCs after double staining for acetylated tubulin (Ac Tub)/y-tubulin **(G)** and Ac Tub/ARL13b **(H)**. Stars indicate cilia. **(I,J)** Quantification of ARL13b^+^/PDGFR-a^+^ cells respectively in proliferation **(I)** and differentiation medium **(J)**. Statistical significance was determined by two-way ANOVA and Bonferroni *post-hoc* test was employed for multiple comparisons. Data are presented as mean ± error of the mean (SEM) and asterisks indicate: **p < 0.01. Scale bar is 10 μm in **(A–C)**, 5 μm in **(D)**, 10 μm in **(G,H)**.

Analyses were performed with GraphPad Prism 7 Software (GraphPad Software, USA). Oligodendroglial cells distribution (qualitative variables) in Figures 1E,F was analyzed using Chi- Square test.

For other experiences, Student’s *t*-test or ANOVA (One- or Two-way) followed by Bonferroni *post-hoc* test were employed. Data are presented as mean ± standard error of the mean (SEM). The list of the applied statistical tests is included in Supplementary Table 4.

## Results

### The proportion of ciliated oligodendrocyte precursor cells depends on culture medium

We first studied the occurrence of a PC on oligodendroglial purified cells grown in two different culture environments, respectively, called proliferation and differentiation medium, based on their composition. Cells were classified in four differentiation stages according to their morphological complexity, markers expression (PDGFR-a, CC1 and MBP) and eGFP fluorescence intensity as shown in Figures 1A–D.

Analysis of cells distribution showed predominant OPCs and pre-oligodendrocytes at the three times of culture in proliferation medium (Figure 1E), while in differentiation medium (Figure 1F), immature and mature oligodendrocytes appeared gradually, in a time-dependent manner.

PCs were stained by acetylated tubulin (Figure 1G) and ARL13b (Figure 1H). Since all acetylated tubulin PCs were also ARL13b^+^ (Figure 1H), we used ARL13b as cilium marker for quantification. Only OPCs and pre-oligodendrocytes had a PC and the percentage of ciliated PDGFR-a^+^ cells increased with time in proliferation medium (Figure 1I) but not in differentiation medium (Figure 1J).

### Culture medium impacts cilium response to Lithium and forskolin

To study the plasticity of PC in oligodendroglial cells, we treated oligodendroglial cell line 158N and murine purified OPCs cultures with LiCl and forskolin, two chemicals able to elongate PC in some cell types.

**FIGURE 2:**
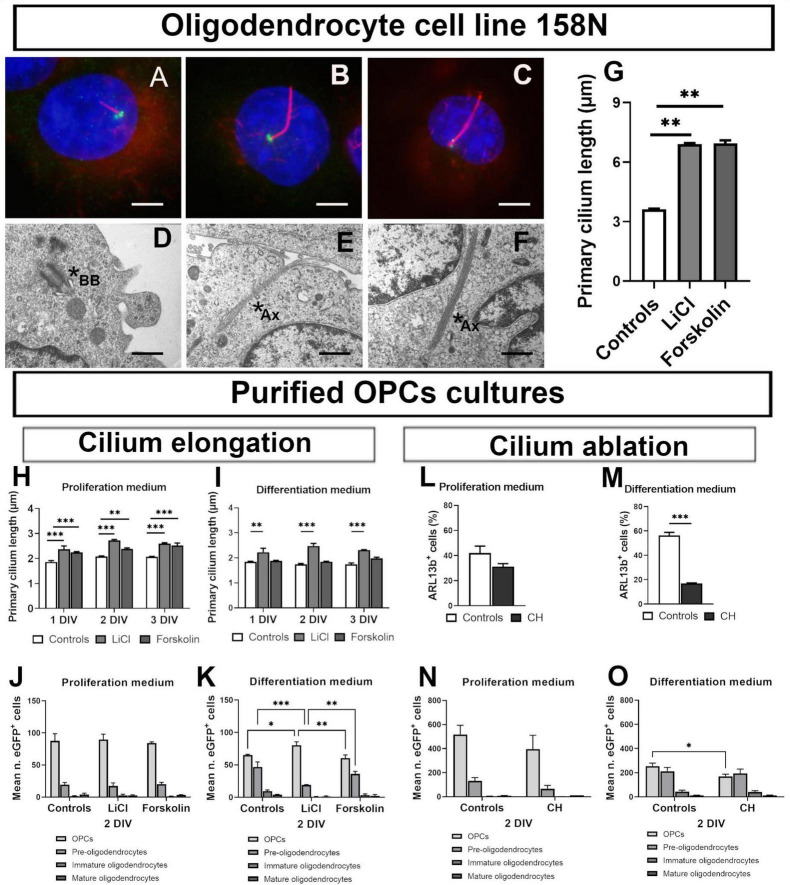
Primary cilium plasticity in oligodendroglial cell line 158N and purified OPCs cultures. **(A–F)** Cilium in 158 cell line. **(A–C)** Representative photomicrographs of cilia in controls **(A)**, LiCl **(B)** and forskolin **(C)** treated 158N cells. Primary cilium was stained by acetylated tubulin (Ac Tub) and basal body by y-tubulin. Scale bar: 5 μm. **(D–F)** Representative ultrastructural PC morphology. Mother centriole beginning to extend a short axoneme associated to its membrane vesicle in the cytoplasm **(D)** and long axonemes in LiCl treated cells **(E,F)**; scale bar: 300 nm. Stars shown basal body (BB) in **(D)** and ciliary axoneme (Ax) in **(E,F)**. **(G)** Both Lithium and forskolin lengthened cilia in 158N cells (One-way ANOVA followed by Bonferroni *post-hoc* test; **p < 0.01; *n* = 3). **(H–O)** Cilium in primary oligodendroglial cell cultures. **(H–K)** Effect of lithium chloride and forskolin. In proliferation medium, both lithium and forskolin lengthened cilia **(H)**. In differentiation medium only lithium was able to elongate primary cilium **(I)** at the tree times studied (one-way ANOVA followed by Bonferroni *post-hoc* test; **p < 0.01, ***p < 0.001. *n* = 3). **(J,K)** The distribution of eGFP^+^ cells at 2 DIV of culture was not modified in proliferation medium. **(J)** In differentiation medium, lithium maintains cells in an immature state while forskolin had no effect. **(K)** Statistical significance was determined by two-way ANOVA and Bonferroni *post-hoc* test was employed for multiple comparisons. Data are presented as mean ± error of the mean (SEM) and asterisks indicate: *p < 0.05, **p < 0.01, ***p < 0.001. **(L–O)** Effect of chloral hydrate (CH). CH did not ablate PDGFR-a^+^ cells PC in proliferation medium **(L)** but only in differentiation medium **(M)** (student’s *t*-test; ***p < 0.001). **(N,O)** Effect of CH on eGFP^+^ cells distribution in proliferation and differentiation media at 2 DIV of culture. CH did not modify cells distribution in proliferation medium **(N)**; in differentiation medium **(O)**, CH treatment reduced the number of cells in the less differentiated stages. Statistical significance was determined by Two-way ANOVA and Bonferroni *post-hoc* test was employed for multiple comparisons. Data are presented as mean ± error of the mean (SEM) and asterisks indicate: *p < 0.05.

In cell line 158N, cilia were clearly identified by acetylated tubulin and were longer than those of primary oligodendroglial cells. Figures 2A–F illustrates the effect of treatment on cilium length observed by immunofluorescence and electron microscopy. In these cells, LiCl and forskolin importantly increased PC length (Figure 2G).

In contrast, PC response in primary oligodendroglial cells cultures depended on culture conditions. Both, LiCl and forskolin elongated PC in proliferation medium (Figure 2H) but only Lithium acted in differentiation medium (Figure 2I).

The distribution of cells at 2 DIV was not modified by Lithium and forskolin treatment in proliferation medium (Figure 2J) while in differentiation medium, forskolin had no effect and Lithium maintained cells in a less differentiated state as compared to controls (Figure 2K).

Further we treated purified oligodendrocytes cultures with Chloral Hydrate (CH), a chemical able to ablate PC in some cell types. CH reduced the proportion of ciliated cells only in differentiation medium (Figures 2L,M). In proliferation medium, as expected for an anti-mitotic drug, the total number of cells was reduced by CH treatment and the cells were maintained in an immature stage (Figure 2N) while in differentiation medium, CH reduces the number of cells less differentiated (Figure 2O).

### Oligodendrocyte precursor cells primary cilium heterogeneity in corpus callosum and cerebral cortex during development

To attest the relevance of data obtained *in vitro*, showing that OPCs differently develop a PC depending on their environment, we studied PC in oligodendroglial cells in white (corpus callosum) and gray (cerebral cortex) matters during post-natal myelination from P2 to P30.

**FIGURE 3:**
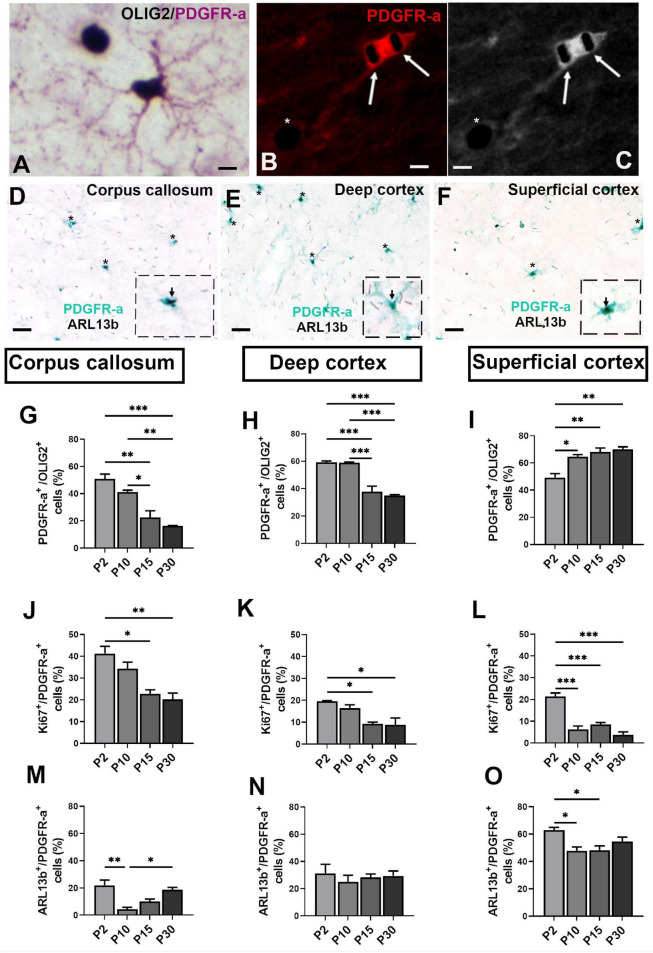
Ciliated oligodendroglial cells distribution in corpus callosum and cortex during mouse post-natal development. **(A,B)** Representative images of double staining PDGFR-a/OLIG2 **(A)** and Ki67/PDGFR-a **(B,C)**, respectively. Arrows in **(B)** indicate Ki67^+^ nuclei staining in black (SG HRP Substrate) in a PDGFR-a^+^ cell (red fluorochrome) and asterisk shown a Ki67^+^ nucleus in a PDGFR-a^–^ cell. **(C)** Black and white image of **(B)**. **(D–F)** Representative image of ciliated PDGFR-a^+^ cells in mouse corpus callosum, deep and superficial cortex at post-natal day 15. Asterisks indicate primary cilia stained with ARL13b in black (SG HRP Substrate) in PDGFR-a^+^ cells stained by a green HRP Substrate (HistoGreen). In the dotted squares, high magnification of a PDGFR^+^/ARL13b^+^ cells. **(G–O)** Quantification of PDGFR-a^+^ **(G–I)**, Ki67^+^ **(J–L)** and ARL13b^+^ cells **(M–O)** in corpus callosum, deep and superficial cortex. Groups were compared using one-way ANOVA followed by Bonferroni *post-hoc* test. Data are presented as mean ± error of the mean (SEM) and asterisks indicate: *p < 0.05, **p < 0.01, ***p < 0.001; the values for n are available in Supplementary Table 4. Scale bar is 10 μm in **(A–C)** and 20 μm in **(D–F)**. The inspected cells for the evaluation of ciliated PDGFR-a^+^ percentage was 40–50 cells repeated in three different slices for each animal and each anatomical region studied at the different ages.

OPCs and proliferating OPCs during development were identified by PDGFR-a/OLIG2 (Figure 3A) and Ki67/PDGFR-a (Figures 3B,C) double staining. *In vivo* as was the case in cultures, only PDGFR-a^+^ cells had an ARL13b^+^ cilium (Figures 3D–F).

As expected, the proportion of PDGFR-a^+^ cells decreased in corpus callosum and deep cortex (Figures 3G,H) from P2 to P30 in parallel with the progressive myelination. In contrast, in superficial cortex, this proportion increased continuously from P2 to P30 (Figure 3I). The rate of proliferation of PDGFR-a^+^ cells was highest in corpus callosum and decreased during post-natal myelination in both corpus callosum and cortex (Figures 3J–L).

The proportion of ciliated PDGFR-a^+^ cells and their development differed between corpus callosum and cortex. This proportion was lowest in corpus callosum and highest in superficial cortex (Figures 3M–O). In corpus callosum, the proportion sharply decreased from P2 to P10 followed by a progressive increase until P30 (Figure 3M), as was also observed in superficial cortex (Figure 3O). In deep cortex, the proportion of ciliated PDGFR-a^+^ cells did not vary during post-natal development (Figure 3N).

### Characterization of ciliated oligodendrocyte precursor cells during demyelination/remyelination in cuprizone mouse model

**FIGURE 4:**
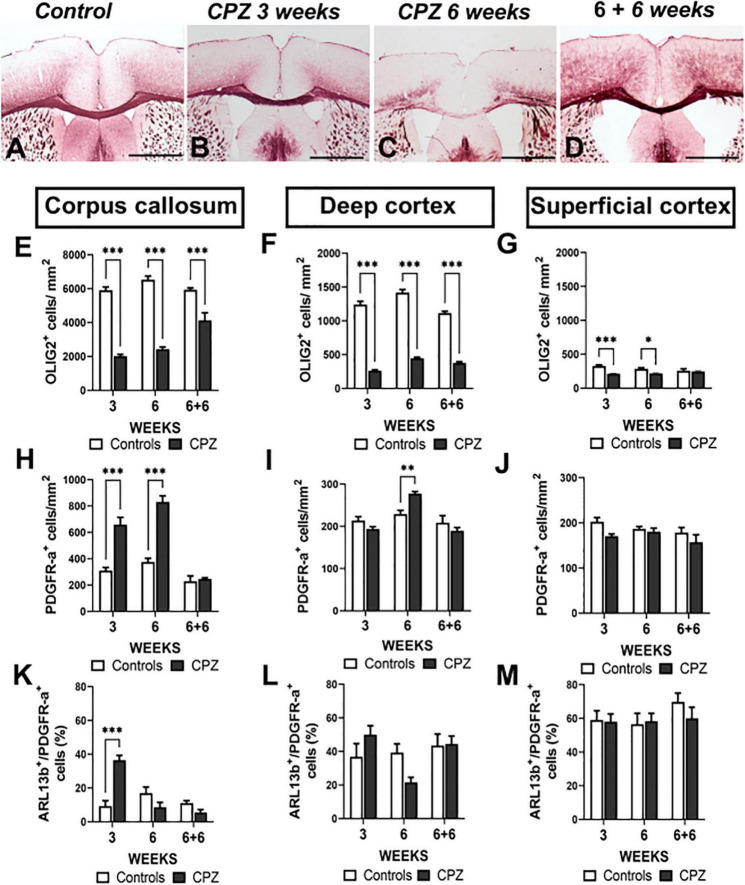
Ciliated oligodendroglial cells distribution in corpus callosum and cortex during demyelination in cuprizone mouse model. **(A–D)** Representative images of MBP immunostaining in controls **(A)**, after 3 **(B)** and 6 weeks **(C)** of cuprizone (CPZ) administration and 6 weeks after treatment withdrawal **(D)**. **(E–M)** Quantification of OLIG2^+^ **(E–G)**, PDGFR-a^+^ **(H–J)** and ARL13b^+^ cells **(K–M)** in corpus callosum, superficial cortex and deep cortex during cuprizone induced demyelination. Groups were compered using Two-way ANOVA followed by Bonferroni *post-hoc* test. Data are presented as mean ± error of the mean (SEM) and asterisks indicate: *p < 0.05, **p < 0.01, ***p < 0.001. The values for n are available in Supplementary Table 4. Scale bar is 500 μm in **(A–D)**. The inspected cells for the evaluation of ciliated PDGFR-a^+^ percentage was 30–50 cells repeated in three different slices for each animal and each anatomical region studied.

Myelin loss started at 3 weeks and became severe at 6 weeks of cuprizone treatment (Figures 4A–D) as confirmed by important loss of OLIG2^+^ cells in corpus callosum and deep cortex (Figures 4E–G). After 3 weeks of cuprizone treatment, MBP immunostaining remained strong due to the presence of MBP^+^ debris from myelin and oligodendrocytes in corpus callosum and cortex (Supplementary Figure 1). Six weeks after the end of cuprizone treatment, the number of oligodendroglial cells was recovered in superficial cortex (Figure 4G), partially recovered in corpus callosum (Figure 4E) while remaining very low in deep cortex (Figure 4F).

As expected in the cuprizone model, we found strong astrocytic and microglial reactions in corpus callosum and deep cortex; 6 weeks after the end of treatment, these reactions were attenuated. No astrocytic and microglial reactions were observed in superficial cortex (Supplementary Figure 2).

The number of PDGFR-a^+^ cells was increased in corpus callosum at early (3 weeks) and late (6 weeks) demyelination stages (Figure 4H), while in deep cortex, a small increase in the number of PDGFR-a^+^ cells was observed only at 6 weeks of cuprizone treatment (Figure 4I). In superficial cortex the number of PDGFR-a^+^ cells did not differ from that of controls at any time of treatment (Figure 4J). In the three regions, 6 weeks after the end of cuprizone treatment, there were no differences in the number of PDGFR-a^+^ cells in treated and control mice (Figure 4H–J).

Interestingly, the changes in ciliated cells (ARL13B^+^/PDGFR-a^+^) population did not match that of PDGFR-a^+^ cells. We only observed a sharp increase of ciliated cells in corpus callosum at 3 weeks of demyelination (Figure 4K). At later stages in corpus callosum and in deep and superficial cortex during the different stages, the proportion of ciliated cells did not differ in treated mice as compared to controls (Figure 4K–M).

## Discussion

The main aim of this investigation was to study PC in oligodendrocyte lineage cells during post-natal development and in a mouse model of demyelination/remyelination. Our results confirm that only PDGFR-a^+^ cells are ciliated and reveal heterogeneity in the frequency of cilium presence on OPCs, depending on culture conditions *in vitro* and cerebral regions *in vivo*. We showed the plasticity of oligodendroglial PC length in response to different drugs. Since dramatic morphological changes of oligodendroglia are correlated with the progression of differentiation and sensitivity to environmental cues (Barateiro and Fernandes, 2014), we analyzed oligodendroglial PC in different culture conditions and in cerebral regions with different myelination levels The dynamic of OPCs proliferation and differentiation *in vitro* intimately correlates with the components of culture media (Lü et al., 2008). The mitogen factors, PDGF and Shh (Merchán et al., 2007) maintain cells in a proliferating state, while when thyroid hormones are added, proliferation and differentiation simultaneously occur, recapitulating *in vivo* development (Durand and Raff, 2000; Tang et al., 2000; Franco et al., 2008). The proportion of ciliated cells in culture increased in proliferation medium, where proliferation index was high, while in differentiation medium, the proportion did not vary. This observation reinforce the role of oligodendroglial cilium in proliferation, as suggested by Cullen et al. (2020). However, this result also suggests that PC could be implicated in transition from proliferative to differentiating stages. Under proliferative environment, many ciliated cells are generated awaiting for differentiation signals This hypothesis may explain why in the absence of proliferation factors in differentiation medium, the proportion of ciliated cells is higher than in proliferation medium after 1 day of culture but does not change over time.

One point concerning OPCs *in vitro* needs to be raised: in our cultures, cells from the whole cerebral cortex are cultured, the subventricular zone (SVZ) as well as parenchymal OPCs. We observed *in vivo* heterogeneity in the presence of a PC in OPCs depending on cerebral regions, longitudinal single cells studies may help refining our understanding on oligodendroglial PC.

Our results suggest the PC as a new marker of OPCs heterogeneity regarding the difference between cerebral cortex and corpus callosum in ciliated OPCs. We found different number of OPCs available for myelination and different frequencies in the presence of PC in different regions with different myelin levels. This is in keeping in mind with recent studies pointing to a molecular and functional heterogeneity of OPCs, attributed to their developmental origin (Foerster et al., 2019; Spitzer et al., 2019), their localization in white and gray matter (Foerster et al., 2019; Spitzer et al., 2019; Winkler and Franco, 2019) and their transduction signaling for proliferation and differentiation (Spitzer et al., 2019; Winkler and Franco, 2019).

In corpus callosum, the most myelinated region, we observed the lowest frequency of ciliated OPCs. This frequency is much lower than that reported by Cullen et al. (2020); indeed we only considered elongated PC and not punctate PC because the latter may be remnants of PC (Paridaen et al., 2013) with either capacity to reassemble a fully developed PC or to disappear with centrosome loss. During post-natal development, the number of ciliated OPCs sharply decreased in corpus callosum from P2 to P10 stage, concomitantly with the onset of myelination. The same situation occurred after cuprizone treatment: following a sharp rise in ciliated OPCs at 3 weeks treatment, the frequency was decreased in correlation with the onset of myelination (Gudi et al., 2009). This raises the question of OPCs origin: during the first post-natal week, OPCs present in the corpus callosum are deriving most from the dorsal SVZ, migrating within corpus callosum and deep cortex (Kessaris et al., 2006), although ventrally derived OPCs are still present. Later, corpus callosum OPCs derive mainly from parenchymal OPCs proliferation. During acute phase of demyelination, OPCs originate from both SVZ and corpus callosum parenchyma (Brousse et al., 2015). Then, two hypothesis may be proposed for the sharp rise of ciliated OPCs at 3 weeks of CPZ treatment: pre-existing parenchymal OPCs with remnants of PC in the cytoplasm could reassemble a PC and proliferate; however, our observation of the highest concentration of ciliated OPCs above the SVZ rather favors a SVZ origin. Proliferating OPCs migrating in the corpus callosum could there encounter a favorable environment for differentiation. Previous studies showed that white matter is more favorable for OPCs differentiation than gray matter and during demyelination in CC (Viganò et al., 2013), cells expressing the morphogen Shh have been identified (Ferent et al., 2013). Moreover, astrocytes are involved in OPCs proliferation by secretion of growth factors (Houben et al., 2020; Rawji et al., 2020).

In deep cortex, where adult myelination is important as compared to superficial cortex, we did not observe modifications in the proportion of ciliated OPCs during post-natal development and demyelination/remyelination. Most studies focus on corpus callosum and very little is known about OL and myelination/remyelination in cortex. However, recent studies suggest that developmental OPCs differentiation and myelination occur earlier in cortex as compared to corpus callosum (Hilscher et al., 2022). We observed that the increase in OPCs due to demyelination was delayed in deep cortex as compared to corpus callosum. Other authors also mentioned a delay in demyelination between cortex and corpus callosum (Gudi et al., 2009). At our knowledge, it is not fully known to which extent both origins of new OPCs contribute to deep cortex remyelination, and which are the migrating capacities of SVZ-derived OPCs during cuprizone-induced demyelination. Further studies will be needed to determine if the ciliated OPCs we observed in corpus callosum during early demyelination participate to cortex remyelination or if there are two separate pools, region-specific, of remyelinating OPCs. Our results also suggest a limitation in ciliated OPCs for differentiation in deep cortex compared to corpus callosum. Indeed, in corpus callosum the sharp increase in ciliated OPCs followed by a sharp decrease suggests that the cells underwent differentiation before a steady state in proliferation/differentiation of parenchymal OPCs. However, less pronounced microgliosis and astrogliosis could also reduced OPCs recruitment and differentiation (Kotter et al., 2001; Buschmann et al., 2012; Sen et al., 2022).

In superficial cortex, where myelination and OPCs proliferation are weak, the remyelination is efficient as previously observed by Orthmann-Murphy et al. (2020), we found the highest level of ciliated OPCs. This region has also the lowest astrogliosis and microgliosis following cuprizone treatment. It has been suggested that the inability to fully recover myelin in deep cortex results from inhibition of myelination, in part due to inflammation (Baxi et al., 2017; Kirby et al., 2019). However, the difference in remyelination between superficial and deep cortex could also result from the availability in new OPCs, resident OPCs being sufficient in the low myelinated superficial cortex. During homeostasis, resident OPCs are perhaps in a non-proliferating non-differentiating favorable environment, explaining the high frequency of ciliated OPCs; these ciliated OPCs could be immediately receptive to differentiating cues due to the presence of PC.

Cells can regulate several of their functions by PC morphological and functional modifications. PC length can be modulated by drugs such as Lithium salts in mesenchymal cells or neurons *in vitro* and *in vivo* (Miyoshi et al., 2009, 2011; Thompson et al., 2016; Oliazadeh et al., 2017; Spasic and Jacobs, 2017) or by cAMP (Besschetnova et al., 2010; Abdul-Majeed et al., 2012). This prompted us to test the possibility to modify oligodendroglial PC. Forskolin only elongated PC in proliferation medium. cAMP signaling is complex in cells with a PC; recent studies revealed that ciliary and cytoplasmic cAMP constitute two pools which can be differently produced and can differently act on cell biology and cilium length (Pazour and Witman, 2003; Anvarian et al., 2019; Nachury and Mick, 2019; Hansen et al., 2020, 2022; Truong et al., 2021; Wachten and Mick, 2021). Moreover, cAMP induces oligodendroglial differentiation (Raible and McMorris, 1993; Ghandour et al., 2002) but this effect was not observed here, probably because of the short time exposure to forskolin. Oppositely, PC length increased in OPCs in response to Lithium independently from culture environment without modifying the number of ciliated OPCs (Ou et al., 2009). Lithium maintained oligodendroglial cells in an immature state in both proliferation and differentiation media. However, in a previous study, Meffre et al. (2015) observed a morphological differentiation of oligodendrocytes attested by a 6-fold increase in MBP mRNA under Lithium treatment in primary mixed glial cells cultures. The difference in results may be explained by culture conditions: Meffre et al. (2015) used primary culture where oligodendrocytes lie on astrocytes bed-layer. It has been recently observed that astrocytes are also direct targets of Lithium (Rivera and Butt, 2019) and they support OPCs differentiation by secreting soluble growth factors or by establishing physical contacts (Nutma et al., 2021). In contrast, as our cultures are oligodendrocytes enriched at 90%, Lithium action may rather be direct on oligodendroglial cells.

To reinforce a possible correlation between PC elongation and OPCs lithium-induced differentiation impairment, we ablated PC using Chloral Hydrate (Chakrabarti et al., 1998; Praetorius and Spring, 2003; Overgaard et al., 2009). Chloral hydrate was not able to decrease the number of ciliated cells in proliferation medium and the cells were maintained in undifferentiated stage. However, in presence of differentiation factors, the absence of cilium had a slight effect in favor of differentiation This effect is not due to proliferation impairment since proliferation was not affected by Chloral Hydrate in differentiation medium. Cullen et al. (2020) showed a reduction of OPCs proliferation using a Cre lox approach to prevent cilia assembly. However, the effects on OPCs differentiation were not reported. Recently, several authors compared the effects of inhibiting PC by either small interfering RNAs for the PC protein IFT88, as used by Cullen et al. (2020) or PC specific inhibitor (chloral hydrate) and observed similar effects on bone and cartilage cells physiology (Martín-Guerrero et al., 2020; Shi et al., 2020). The plasticity of PC in oligodendroglial cells is a complex mechanism. Although our results need to be reinforced to highlight the relationships between cilium length modifications and OPCs differentiation, they suggest a potential effect of Lithium in OPCs proliferation/differentiation through PC modulation.

Recently, several studies showed PC dysfunctions in the pathogenesis of neurodegenerative (Karunakaran et al., 2020) and psychiatric diseases (Muñoz-Estrada et al., 2018) and pointed PC as a new marker of disease and as a potential target for new drugs development. Since, Lithium has beneficial impact on peripheral remyelination (Makoukji et al., 2012; da Silva et al., 2014; Fang et al., 2016; Kuffler, 2021), in a preliminary study (data not shown), we treated mice with Lithium during the 6 weeks period after withdrawal of cuprizone; in deep cortex where remyelination is delayed as compared to corpus callosum, myelin deficit was even more pronounced as compared to Lithium-untreated mice. This can be explained by our *in vitro* results where Lithium maintained oligodendroglial cells in an undifferentiated state.

However, Lithium treatment seemed to induce a shift to higher values of both myelinated axons and fibers diameters. These preliminary observations suggest differential roles of Lithium, dependent on oligodendroglial stage of differentiation.

In conclusion, our results suggest the PC as a new marker of heterogeneity of oligodendroglial lineage cells and present new data, in CNS developmental myelination and remyelination. Although we are aware of some limitations in our study which remains on a descriptive level, it opens a field of research where molecular approaches could answer why PC occurs only in a subset of OPCs, how PC in these OPCs maintain them apart from other OPCs and what are the real functions of PC during development and in demyelination/remyelination.

## Data availability statement

The raw data supporting the conclusions of this article will be made available by the authors, without undue reservation.

## Ethics statement

This animal study was reviewed and approved by the Comité d’Éthique en Matière d’Expérimentation Animale de Strasbourg (CREMEAS).

## Author contributions

GD performed the experiments, analyzed the data, and participated to the experimental design under the supervision of NB. KB and BS contributed to the experiments, data analysis, and experimental design. MG and MA contributed to the experimental design and edited the manuscript. JG performed the material preparation and experiments. NB conceived the idea. GD and NB wrote and edited the manuscript together. All authors approved the final manuscript.
