# Supplementary material for: Oligodendroglial primary cilium heterogeneity during development and demyelination/remyelination
Source: Front Cell Neurosci. 2022 Nov 24;16:1049468. doi: 10.3389/fncel.2022.1049468 (PMC9729284; doi:10.3389/fncel.2022.1049468)
Supplement: Supplementary file 1 [file Data_Sheet_1.pdf]

Oligodendroglial primary cilium heterogeneity during development and demyelination/remyelination

Giada Delfino* [1,2,3], Karelle Bénardais [1,2,3,4] , Julien Graff [2,3], Brigitte Samama [1,2,3,4], Maria Cristina Antal [1,2,3,4], M. Said Ghandour [1,3], Nelly Boehm [1,2,3,4]

[1] ICube laboratory UMR 7357, team IMIS, Strasbourg, France
[2] Institut d'Histologie, Service Central de Microscopie Electronique, Faculté de Médecine, Université de Strasbourg, France
[3]Fédération de Médecine Translationnelle de Strasbourg (FMTS), Strasbourg, France
[4] Hôpitaux Universitaires de Strasbourg, France

Corresponding author: Giada Delfino giada.delfino@inserm.fr

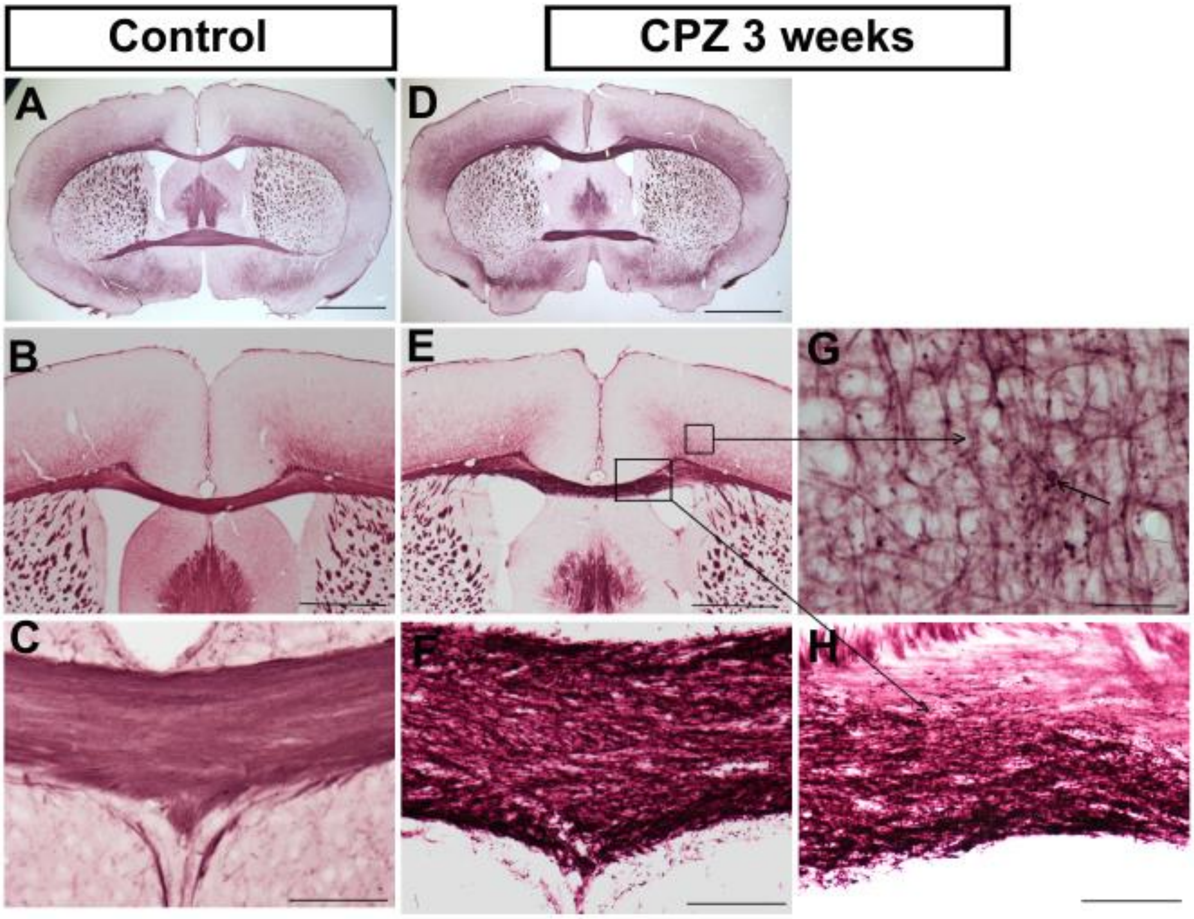

Supplementary Figure 1. MBP immunostaining after 3 weeks of cuprizone induced demyelination in mouse. Representative images of a control **(A-C)** and a cuprizone treated mouse **(D-H)**. Images in **(G)** and **(H)** are respectively cortex and corpus callosum high magnification of **(E)**. Arrows in **(G)** and **(H)** show very high MBP^{+} cellular debris. Scale bar is 2mm in **(A, D)**, 500µm in **(B, E),** 100µm in **(C, G, F, H)**.

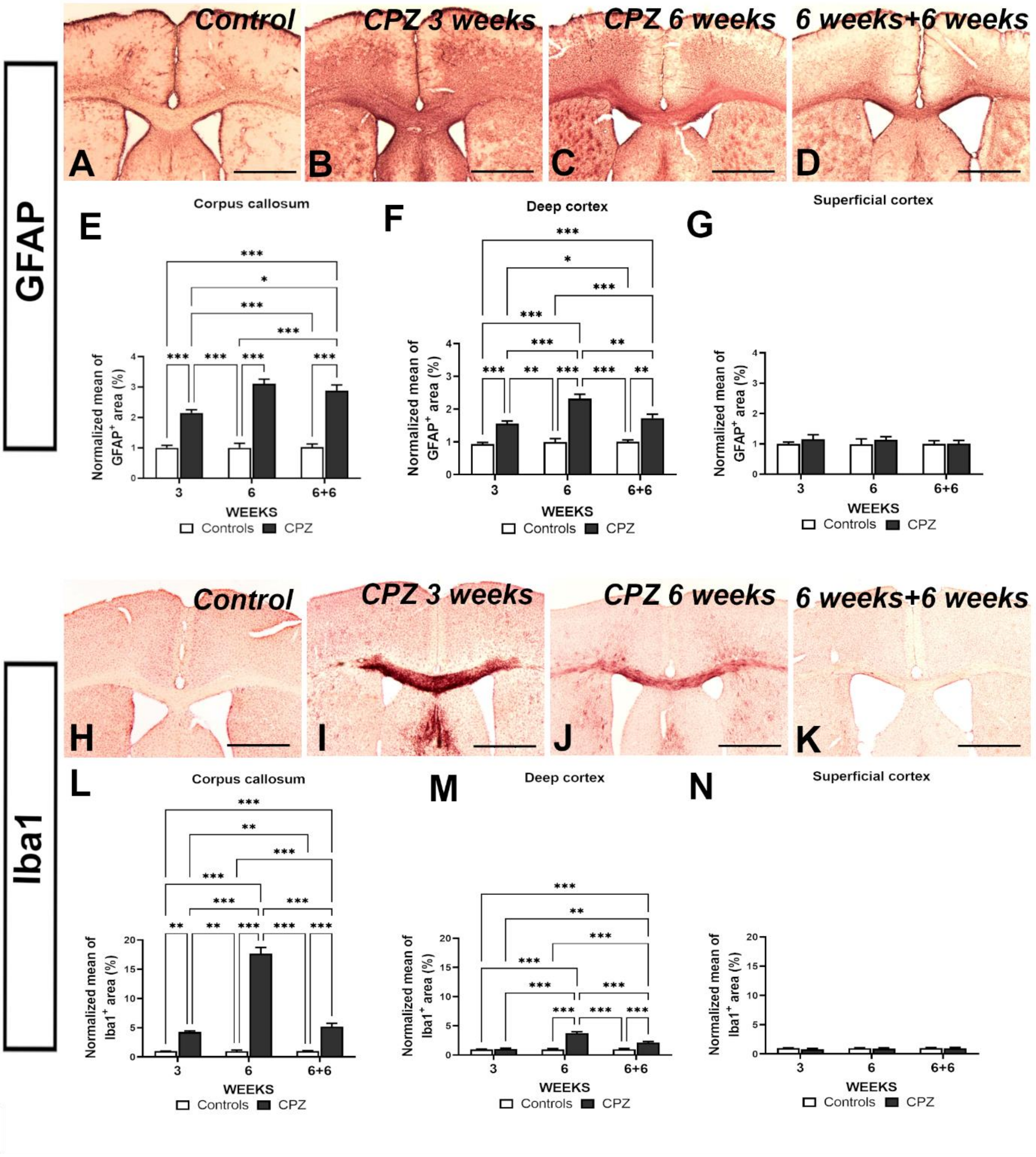

Supplementary Figure 2. Astrocytic and microglial activation during cuprizone induced demyelination and remyelination. **(A-D)** Representative images of GFAP immunostaining in controls **(A),** after 3 weeks **(B)** and 6 weeks **(C)** of cuprizone (CPZ) administration and after 6 weeks of treatment withdrawal **(D)**. **(E-G)** Graphs show the normalized mean of $GFAP^+$ area in cuprizone treated animals compared to controls in corpus callosum, superficial cortex and deep cortex. **(G-I)** Representative images of $Iba1^+$ immunostaining in controls **(H),** after 3 weeks **(I)** and 6 weeks **(J)** of CPZ administration and after 6 weeks of treatment withdrawal **(K)**. **(L-N)** Graphs show the normalized mean of $Iba1^+$ area in cuprizone treated animals

compared to controls in corpus callosum, superficial cortex and deep cortex. Groups were compared using Two-way ANOVA followed by Bonferroni post hoc test. Data are presented as mean ± error of the mean (SEM) and asterisks indicate: **$p<0.01$ ***$p<0.001$ ****$p<0.0001$. The values for n are available in Supplementary Table 4. Scale bar is 500µm in (A-D; H-K).
