# Supplementary material for: Oligodendroglial primary cilium heterogeneity during development and demyelination/remyelination
Source: Front Cell Neurosci. 2022 Nov 24;16:1049468. doi: 10.3389/fncel.2022.1049468 (PMC9729284; doi:10.3389/fncel.2022.1049468)
Supplement: Supplementary file 2 [file Data_Sheet_2.pdf]

Oligodendroglial primary cilium heterogeneity during development and demyelination/remyelination

Giada Delfino* [1,2,3], Karelle Bénardais [1,2,3,4] , Julien Graff [2,3], Brigitte Samama [1,2,3,4], Maria Cristina Antal [1,2,3,4], M. Said Ghandour [1,3], Nelly Boehm [1,2,3,4]

[1] ICube laboratory UMR 7357, team IMIS, Strasbourg, France
[2] Institut d'Histologie, Service Central de Microscopie Electronique, Faculté de Médecine, Université de Strasbourg, France
[3] Fédération de Médecine Translationnelle de Strasbourg (FMTS), Strasbourg, France
[4] Hôpitaux Universitaires de Strasbourg, France

Corresponding author: Giada Delfino giada.delfino@inserm.fr

Table 1. Proliferation and differentiation medium composition.

Proliferation medium	Differentiation medium
• DMEM (1X) Glutamax (Gibco, France)<br><br>• FBS 0.5%<br><br>• Penicillin/streptomycin 0.5%<br>• OPCDS (1/100e) (ScienCell Research Laboratories, Carlsbad, USA)	• DMEM (1X) Glutamax (Gibco, France),<br><br>• FBS 0.5%<br><br>• Penicillin/streptomycin 0.5%<br>• Bovine serum albumin (500 µg ml^{-1})<br><br>• Transferrin (50 µg ml^{-1}),<br>• Insulin (5 µg ml^{-1})<br>• Progesterone (60 ng ml^{-1})<br>• Putrescine (16 µg ml^{-1})<br>• Triiodothyronine (0.4 µg ml^{-1})<br>• B27 supplement 2%

Table 2. Primary antibodies list.

Primary antibodies	Host	Dilution	Company
ARL13b	Rabbit	1/1000e	Proteintech
y- tubulin	Rabbit	1/1000e	Sigma
GFAP	Rabbit	1/2000e	DAKO
Iba1	Rabbit	1/2000e	WAKO
OLIG2	Rabbit	1/500e	Merck Millipore
PDGFR-a	Rabbit	1/500e	Cell Signaling
Ki67	Rabbit	1/200e	Thermo Fishier Scientific
Acetylated tubulin	Mouse	1/2000e	Sigma
Adenomatous polyposis coli (APC) [CC1]	Mouse	1/1000e	Merck Millipore
y-tubulin	Mouse	1/1000e	Sigma
MBP	Mouse	1/1000e	Biolegend

Table 3. Secondary antibodies list.

Secondary antibodies	Dilution	Company
Anti rabbit Alexa Fluor 488	1/400e	Invitrogen
Anti rabbit Alexa Fluor 568	1/400e	Invitrogen
Anti mouse Alexa Fluor 488	1/400e	Invitrogen
Anti rabbit Alexa Fluor 568	1/400e	Invitrogen
Anti rabbit biotinylated	1/200e	Vector
Anti mouse biotinylated	1/200e	Vector
