# Supplementary material for: Oligodendroglial primary cilium heterogeneity during development and demyelination/remyelination
Source: Front Cell Neurosci. 2022 Nov 24;16:1049468. doi: 10.3389/fncel.2022.1049468 (PMC9729284; doi:10.3389/fncel.2022.1049468)
Supplement: Supplementary file 3 [file Data_Sheet_3.PDF]

Table 4. Statistics

Figure	Applied test	n	P value	Statistics	Post hoc analyses
**Fig. 1E**	Chi-square test	1 DIV = 3 slices<br>2 DIV = 3 slices<br>3 DIV = 3 slices	P<0.001	**Chi-square, df**<br><br>83.26, 6	
**Fig. 1F**	Chi-square test	1 DIV = 3 slices<br>2 DIV = 3 slices<br>3 DIV = 3 slices	P<0.001	**Chi-square, df**<br>242.9, 6	
**Fig. 1I**	Two-way ANOVA	1 DIV = 3 slices<br>2 DIV = 3 slices<br>3 DIV = 3 slices	**Time**<br>P<0.001<br>**Cell stage**<br>P<0.01<br>**Interaction**<br>n.s.	**Time**<br>F (2, 12) =14.83<br>**Cell stage**<br>F (1, 12) =10.09<br>**Interaction**<br>F (2, 12) =3.855	Bonferroni Multiple comparisons test
**Fig. 1J**	Two-way ANOVA	1 DIV = 3 slices<br>2 DIV = 3 slices<br>3 DIV = 3 slices	**Time**<br>P<0.05<br>**Cell stage**<br>P<0.05<br>**Interaction**<br>n.s.	**Time**<br>F (2, 12) =4.180<br>**Cell stage**<br>F (1, 12) =5.754<br>**Interaction**<br>F (2, 12) =0.4536	Bonferroni Multiple comparisons test
**Fig. 2G**	One-way ANOVA	1 DIV = 3 slices<br>2 DIV = 3 slices<br>3 DIV = 3 slices	P<0.001	F = 469.3	Bonferroni Multiple comparisons test
**Fig. 2H**	Two-way ANOVA	1 DIV = 3 slices<br>2 DIV = 3 slices<br>3 DIV = 3 slices	**Time**<br>P<0.001<br>**Drug**<br>P<0.001<br>**Interaction**<br>n.s.	**Time**<br>F (2, 18) =15.24<br>**Drug**<br>F (2, 18) =68.70<br>**Interaction**<br>F (4, 18) =1.359	Bonferroni Multiple comparisons test
**Fig. 2I**	Two-way ANOVA	1 DIV = 3 slices<br>2 DIV = 3 slices<br>3 DIV = 3 slices	**Time**<br>n.s<br>**Drug**<br>P<0.001<br>**Interaction**<br>n.s.	**Time**<br>F (2, 18) =0.3057<br>**Drug**<br>F (2, 18) =57.27<br>**Interaction**<br>F (4, 18) =2.463	Bonferroni Multiple comparisons test
**Fig. 2J**	Two-way ANOVA	Controls = 3 slices<br>LiCl = 3 slices<br>Forskolin = 3 slices	**Drug**<br>n.s<br>**Cell stage**<br>P<0.001<br>**Interaction**<br>n.s.	**Drug**<br>F (2, 24) =0.06335<br>**Cell stage**<br>F (3, 24) =231.8<br>**Interaction**<br>F (6, 24) =0.1596	Bonferroni Multiple comparisons test
**Fig. 2K**	Two-way ANOVA	Controls = 3 slices<br>LiCl = 3 slices<br>Forskolin = 3 slices	**Drug**<br>P<0.05<br>**Cell stage**<br>P<0.001<br>**Interaction**<br>P<0.001	**Drug**<br>F (2, 24) =3.910<br>**Cell stage**<br>F (3, 24) =244.8<br>**Interaction**<br>F (6, 24) =7.920	Bonferroni Multiple comparisons test
**Fig. 2L**	Unpaired t test (2-tailed)	Controls = 3 slices<br>CH = 3 slices	n.s	t(4) = 1.787	
**Fig. 2M**	Unpaired t test (2-tailed)	Controls = 3 slices<br>CH = 3 slices	P<0.001	t(4) = 15.37	
**Fig. 2N**	Two-way ANOVA	Controls = 3 slices<br>CH = 3 slices	**Drug**<br>n.s.<br>**Cell stage**<br>P<0.001<br>**Interaction**<br>n.s.	**Drug**<br>F (1, 16) =1.642<br>**Cell stage**<br>F (3, 16) =34.85<br>**Interaction**<br>F (3, 16) =0.6637	Bonferroni Multiple comparisons test

**Fig. 2O**	Two-way ANOVA	Controls = 3 slices<br>CH = 3 slices	**Drug**<br>n.s.<br>**Cell stage**<br>P<0.0001<br>**Interaction**<br>n.s.	**Drug**<br>F (1, 16) =3.182<br>**Cell stage**<br>F (3, 16) =49.33<br>**Interaction**<br>F (3, 16) =1.790	Bonferroni Multiple comparisons test
**Fig. 3D**	One-way ANOVA	P2 = 3<br>P10 = 3<br>P15 = 3<br>P30 = 3	P<0.001	F (3, 8) = 26.89	Bonferroni Multiple comparisons test
**Fig. 3E**	One-way ANOVA	P2 = 3<br>P10 = 3<br>P15 = 3<br>P30 = 3	P<0.001	F (3, 8) = 36.86	Bonferroni Multiple comparisons test
**Fig. 3F**	One-way ANOVA	P2 = 3<br>P10 = 3<br>P15 = 3<br>P30 = 3	P<0.001	F (3, 8) = 15.02	Bonferroni Multiple comparisons test
**Fig. 3G**	One-way ANOVA	P2 = 3<br>P10 = 3<br>P15 = 3<br>P30 = 3	P = 0.003	F (3, 8) = 11.87	Bonferroni Multiple comparisons test
**Fig. 3H**	One-way ANOVA	P2 = 3<br>P10 = 3<br>P15 = 3<br>P30 = 3	P = 0.006	F (3, 8) = 8.926	Bonferroni Multiple comparisons test
**Fig. 3I**	One-way ANOVA	P2 = 3<br>P10 = 3<br>P15 = 3<br>P30 = 3	P<0.001	F (3, 8) = 34.42	Bonferroni Multiple comparisons test
**Fig. 3J**	One-way ANOVA	P2 = 3<br>P10 = 3<br>P15 = 3<br>P30 = 3	P = 0.004	F (3, 8) = 10.77	Bonferroni Multiple comparisons test
**Fig. 3K**	One-way ANOVA	P2 = 3<br>P10 = 3<br>P15 = 3<br>P30 = 3	P = 0.81	F (3, 8) = 0.3163	Bonferroni Multiple comparisons test
**Fig. 3L**	One-way ANOVA	P2 = 3<br>P10 = 3<br>P15 = 3<br>P30 = 3	P = 0.02	F (3, 8) = 6.103	Bonferroni Multiple comparisons test
**Fig. 4E**	Two-way ANOVA	Controls 3weeks = 7<br>CPZ 3weeks = 8<br>Controls 6 weeks= 6<br>CPZ 6 weeks = 8<br>Controls 6+6 = 4<br>CPZ 6+6 = 4	**Time**<br>P<0.001<br>**Treatment**<br>P<0.001<br>**Interaction**<br>P<0.001	**Time**<br>F (2, 31) =14.21<br>**Treatment**<br>F (1, 31) =423.8<br>**Interaction**<br>F (2, 31) =17.72	Bonferroni Multiple comparisons test
**Fig. 4F**	Two-way ANOVA	Controls 3weeks = 7<br>CPZ 3weeks = 8<br>Controls 6 weeks= 6<br>CPZ 6 weeks = 8<br>Controls 6+6 = 4<br>CPZ 6+6 = 4	**Time**<br>P<0.001<br>**Treatment**<br>P<0.001<br>**Interaction**<br>P = 0.006	**Time**<br>F (2, 30) =18.40<br>**Treatment**<br>F (1, 30) =913.2<br>**Interaction**<br>F (2, 30) =6.058	Bonferroni Multiple comparisons test
**Fig. 4G**	Two-way ANOVA	Controls 3weeks = 7<br>CPZ 3weeks = 8<br>Controls 6 weeks= 6<br>CPZ 6 weeks = 8	**Time**<br>n.s.<br>**Treatment**<br>P<0.001<br>**Interaction**<br>P = 0.009	**Time**<br>F (2, 32) =1.389<br>**Treatment**<br>F (1, 32) =27.75<br>**Interaction**<br>F (2, 32) =5.479	Bonferroni Multiple comparisons test

		Controls 6+6 = 4<br>CPZ 6+6 = 4			
**Fig. 4H**	Two-way ANOVA	Controls 3weeks = 7<br>CPZ 3weeks = 8<br>Controls 6 weeks= 6<br>CPZ 6 weeks = 8<br>Controls 6+6 = 4<br>CPZ 6+6 = 4	**Time**<br>P<0.001<br>**Treatment**<br>P<0.001<br>**Interaction**<br>P<0.001	**Time**<br>F (2, 31) =28.39<br>**Treatment**<br>F (1, 31) =53.47<br>**Interaction**<br>F (2, 31) =10.37	Bonferroni Multiple comparisons test
**Fig. 4I**	Two-way ANOVA	Controls 3weeks = 7<br>CPZ 3weeks = 8<br>Controls 6 weeks= 6<br>CPZ 6 weeks = 8<br>Controls 6+6 = 4<br>CPZ 6+6 = 4	**Time**<br>P<0.001<br>**Treatment**<br>n.s.<br>**Interaction**<br>P<0.001	**Time**<br>F (2, 30) =23.30<br>**Treatment**<br>F (1, 31) =0.1798<br>**Interaction**<br>F (2, 30) =10.14	Bonferroni Multiple comparisons test
**Fig. 4J**	Two-way ANOVA	Controls 3weeks = 7<br>CPZ 3weeks = 8<br>Controls 6 weeks= 6<br>CPZ 6 weeks = 8<br>Controls 6+6 = 4<br>CPZ 6+6 = 4	**Time**<br>n.s.<br>**Treatment**<br>P = 0.010<br>**Interaction**<br>n.s.	**Time**<br>F (2, 32) =2.099<br>**Treatment**<br>F (1, 32) =7.411<br>**Interaction**<br>F (2, 32) =1.310	Bonferroni Multiple comparisons test
**Fig. 4K**	Two-way ANOVA	Controls 3weeks = 7<br>CPZ 3weeks = 8<br>Controls 6 weeks= 6<br>CPZ 6 weeks = 8<br>Controls 6+6 = 4<br>CPZ 6+6 = 4	**Time**<br>P<0.001<br>**Treatment**<br>n.s.<br>**Interaction**<br>P<0.001	**Time**<br>F (2, 28) =14.09<br>**Treatment**<br>F (1, 28) =3.708<br>**Interaction**<br>F (2, 28) =25.26	Bonferroni Multiple comparisons test
**Fig. 4L**	Two-way ANOVA	Controls 3weeks = 7<br>CPZ 3weeks = 8<br>Controls 6 weeks= 6<br>CPZ 6 weeks = 8<br>Controls 6+6 = 4<br>CPZ 6+6 = 4	**Time**<br>P = 0.037<br>**Treatment**<br>n.s.<br>**Interaction**<br>P = 0.30	**Time**<br>F (2, 28) =3.727<br>**Treatment**<br>F (1, 28) =0.06353<br>**Interaction**<br>F (2, 28) =4.003	Bonferroni Multiple comparisons test
**Fig. 4M**	Two-way ANOVA	Controls 3weeks = 7<br>CPZ 3weeks = 8<br>Controls 6 weeks= 6<br>CPZ 6 weeks = 8<br>Controls 6+6 = 4<br>CPZ 6+6 = 4	**Time**<br>n.s.<br>**Treatment**<br>n.s.<br>**Interaction**<br>n.s.	**Time**<br>F (2, 28) =0.9700<br>**Treatment**<br>F (1, 28) =0.4274<br>**Interaction**<br>F (2, 28) =0.5495	Bonferroni Multiple comparisons test
**Suppl. Fig. 2E**	Two-way ANOVA	Controls 3weeks = 8<br>CPZ 3weeks = 7<br>Controls 6 weeks= 7<br>CPZ 6 weeks = 7<br>Controls 6+6 = 4<br>CPZ 6+6 = 4	**Time**<br>P<0.001<br>**Treatment**<br>P<0.001<br>**Interaction**<br>P<0.001	**Time**<br>F (2, 31) =8.897<br>**Treatment**<br>F (1, 31) =244.5<br>**Interaction**<br>F (2, 31) =8.739	Bonferroni Multiple comparisons test

**Suppl. Fig. 2F**	Two-way ANOVA	Controls 3weeks = 8<br>CPZ 3weeks = 7<br>Controls 6 weeks= 7<br>CPZ 6 weeks = 7<br>Controls 6+6 = 4<br>CPZ 6+6 = 4	**Time**<br>P<0.001<br>**Treatment**<br>P<0.001<br>**Interaction**<br>P = 0.001	**Time**<br>F (2, 29) =10.80<br>**Treatment**<br>F (1, 29) =118.0<br>**Interaction**<br>F (2, 29) =8.425	Bonferroni Multiple comparisons test
**Suppl. Fig. 2G**	Two-way ANOVA	Controls 3weeks = 8<br>CPZ 3weeks = 7<br>Controls 6 weeks= 7<br>CPZ 6 weeks = 7<br>Controls 6+6 = 4<br>CPZ 6+6 = 4	**Time**<br>n.s.<br>**Treatment**<br>n.s.<br>**Interaction**<br>n.s.	**Time**<br>F (2, 31) =0.1414<br>**Treatment**<br>F (1, 31) =0.8931<br>**Interaction**<br>F (2, 31) =0.1715	Bonferroni Multiple comparisons test
**Suppl. Fig. 2L**	Two-way ANOVA	Controls 3weeks = 8<br>CPZ 3weeks = 7<br>Controls 6 weeks= 7<br>CPZ 6 weeks = 7<br>Controls 6+6 = 4<br>CPZ 6+6 = 4	**Time**<br>P<0.001<br>**Treatment**<br>P<0.001<br>**Interaction**<br>P<0.001	**Time**<br>F (2, 39) =98.20<br>**Treatment**<br>F (1, 39) =343.2<br>**Interaction**<br>F (2, 39) =98.20	Bonferroni Multiple comparisons test
**Suppl. Fig. 2M**	Two-way ANOVA	Controls 3weeks = 8<br>CPZ 3weeks = 7<br>Controls 6 weeks= 7<br>CPZ 6 weeks = 7<br>Controls 6+6 = 4<br>CPZ 6+6 = 4	**Time**<br>P<0.001<br>**Treatment**<br>P<0.001<br>**Interaction**<br>P = 0.001	**Time**<br>F (2, 38) =29.37<br>**Treatment**<br>F (1, 38) =86.88<br>**Interaction**<br>F (2, 38) =29.37	Bonferroni Multiple comparisons test
**Suppl. Fig. 2N**	Two-way ANOVA	Controls 3weeks = 8<br>CPZ 3weeks = 7<br>Controls 6 weeks= 7<br>CPZ 6 weeks = 7<br>Controls 6+6 = 4<br>CPZ 6+6 = 4	**Time**<br>n.s.<br>**Treatment**<br>n.s.<br>**Interaction**<br>n.s.	**Time**<br>F (2, 38) =0.3920<br>**Treatment**<br>F (1, 38) =1.600<br>**Interaction**<br>F (2, 38) =0.3920	Bonferroni Multiple comparisons test
